# Evaluation of Urinary L-FABP as a Tubular Damage Marker in Pediatric Neurogenic Bladder—A Pilot Study

**DOI:** 10.3390/jcm13030736

**Published:** 2024-01-27

**Authors:** Joanna Bagińska, Jan Krzysztof Kirejczyk, Agata Korzeniecka-Kozerska

**Affiliations:** 1 Department of Pediatrics and Nephrology, Medical University of Białystok, 17 Waszyngtona Str., 15-274 Białystok, Poland; agatakozerska@poczta.onet.pl; 2 Faculty of Health Sciences, University of Lomza, 14 Akademicka Str., 18-400 Lomza, Poland; kkirejczyk@wp.pl

**Keywords:** neurogenic bladder, L-FABP, proteinuria

## Abstract

The article aims to find potential biomarker for the detection of tubular damage in pediatric neurogenic bladder (NB) by investigating urinary levels of liver-type fatty acid-binding protein (uL-FABP). This prospective analysis was conducted on two groups: 42 children with NB and 18 healthy children. The uL-FABP concentrations were measured using ELISA methods. The medical charts of the children were examined to determine age, sex, anthropometric measurements, activity assessment using Hoffer’s scale, and renal function parameters. The results revealed that the uL-FABP/creatinine ratio was higher in the study group compared with the reference group, but the difference was not statistically significant (*p* = 0.52, *p* > 0.05). However, the uL-FABP/creatinine ratio exhibited a wider range in NB patients compared to the reference group. NB children with proteinuria and the history of high-grade vesicoureteral reflux (VUR) tended to have the highest uL-FABP concentrations. In conclusion, uL-FABP may be considered a potential tubular damage biomarker in children with NB. Proteinuria and the history of VUR may be the factors influencing the uL-FABP.

## 1. Introduction

Neurogenic bladder (NB) is a heterogeneous entity that may result from a variety of conditions affecting the nervous systems at any level. Among children, myelomeningocele (MMC) is the leading cause, with an estimated prevalence of 1 in 700 live births [1]. Although the incidence of MMC has decreased in recent years thanks to prenatal diagnosis and folic acid supplementation during pregnancy, there are still many children presenting with the signs of NB [2]. At birth, the majority of patients have normal upper urinary tract, but approximately 60% of them will develop renal deterioration due to increased filling detrusor pressures, recurrent urinary tract infections (rUTIs) with or without vesicoureteral reflux (VUR) [3,4]. This process may occur silently and ultimately lead to chronic kidney disease [5]. There is an urgent need for biomarkers for renal deterioration in NB for its prompt diagnosis and treatment control. In this context, current studies have aimed at identifying novel biomarkers that can be used to monitor and predict renal impairment in children with NB [6,7].

Liver-type fatty acid-binding protein (L-FABP) is an intracellular protein that is expressed not only in the liver but also in the intestine, pancreas, stomach, lung, and the proximal tubule of the human kidney. It is found in various organs such as the liver, intestine, pancreas, stomach, lung, and kidney tubules. Numerous studies have demonstrated that L-FABP is a promising biomarker in a wide variety of nephrological disorders, i.e., diabetic kidney disease and acute kidney injury [8,9,10]. Because of its small molecular size, L-FABP is freely filtered and can be easily detected in urine. The small size of L-FABP enables it to be easily detected in urine as it is freely filtered. Urinary L-FABP (uL-FABP) is released in response to tubular damage. Pediatric NB predisposes to upper tract damage, including renal tubules. Upper tract damage, including renal tubules, is a potential consequence of pediatric NB. The lack of proven biomarkers of tubular injury in children with NB pushed us to conduct this study.

## 2. Materials and Methods

### 2.1. Patients

This prospective case–control analysis was performed on 60 children who were divided into two groups. The study group consisted of 42 children with NB after MMC. The University Children’s Hospital in Bialystok, Poland, oversaw the care of all of them in the Department of Pediatrics and Nephrology. They were treated by clean intermittent catheterization. In medical management, regular urodynamic testing was performed to monitor bladder pressures. The study included participants with a high-pressure bladder after they received the appropriate treatment with anticholinergic medications.

The presence of urinary tract infection was excluded based on urinary testing and urine culture. The current infection was disregarded based on a negative C-reactive protein. Additionally, a history of recurrent urinary tract infections (rUTIs) and the presence or history of vesicoureteral reflux (VUR) were recorded. For the diagnosis of VUR, we used the medical records and the gold standard: voiding cystourethrogram (VCUG). Indications for VCUG were rUTIs or investigation of hydronephrosis etiology. International Reflux Classification, using a scale from I to V, was used for VUR grading [11]. We divided the children with NB into 3 groups: 1—without VUR; 2—with present VUR; 3—with a history of VUR.

Hoffer’s scale (HS) was used to define the ambulatory function of MMC patients, classifying community mobility into four categories: 1HS—nonambulatory; 2HS—nonfunctional ambulator; 3HS—household walkers; 4HS—community walkers [12]. In MMC patients, the lesion level was evaluated intraoperatively and radiologically, using a scoring system of 1 to 3 (1—thoracolumbar; 2—lumbosacral; 3—sacral lesion).

The reference group comprised 18 participants who visited a pediatrician for well-child visits and showed no abnormalities in the urinary or nervous systems.

The medical charts of the children were examined to determine age, sex, anthropometric measurements and standard deviation scores WHO (z-scores). In children with MMC, monitoring anthropometric parameters such as body weight, height, and BMI is crucial for assessing their overall health and development. The use of z-scores, calculated based on standard reference values provided by WHO, offers a valuable tool in this regard. z-scores allow to compare an individual child’s measurements to those of a reference population, adjusting for age and sex. The formula for calculating the z-score: (X − m)/SD, where X is the observed value (height, weight, or BMI), m is the mean, and SD is the standard deviation of the distribution corresponding to the reference population, provides a standardized means of evaluating growth parameters in children with MMC. This approach aids in identifying deviations from expected growth patterns and more accurately assess a MMC child’s growth status relative to their peers. Renal function parameters: urinary and serum creatinine and the glomerular filtration rate (GFR) according to the new Schwartz formula were determined.

### 2.2. Biochemistry

L-FABP was measured using first morning void spot urine samples, and the L-FABP ELISA Kit (Cloud-Clone Corp., Houston, TX, USA) was used according to the manufacturer’s instructions. The urinary creatinine (Cr) concentration was used to normalize L-FABP measurements to account for the influence of urinary dilution on concentration. The uL-FABP/Cr ratio in ng/mg creatinine was used to express the biomarker levels in urine.

### 2.3. Statistics

The data were gathered and stored in a Microsoft Excel database. Statistical analysis was performed using Statistica 13.0. (StatSoft Inc., Tulsa, OK, USA). All the studied parameters were analyzed using a nonparametric Mann–Whitney test. Correlations were evaluated using the Spearman test. A significance level of *p* < 0.05 was used.

### 2.4. Ethical Issues

This study was approved by the Ethics Committee of the Medical University of Bialystok which complies with the World Medical Association Declaration of Helsinki regarding ethical conduct of research involving human subjects and/or animals. Patients and their caregivers were enrolled in the study after obtaining informed consent.

## 3. Results

The characteristics of the studied children are presented in Table 1. The median age of the enrolled patients was 11.5 (0.67–18) years. There were no differences in age, sex, z-scores weight to age and z-score BMI to age excluding z-scores height to age. This resulted from a shorter vertebral dimension and malformations of the bone structure due to MMC in the NB group. We found statistically significant differences in urine, serum creatinine concentration, and eGFR Schwartz between the studied groups.

Comparison of the uL-FABP and uL-FABP/Cr ratio is presented in Table 2. As shown, children with NB had an elevated median uL-FABP/Cr ratio in comparison with the reference group, but the differences were not statistically significant (*p*= 0.52, *p* > 0.05). However, the range of the uL-FABP/Cr ratio was wider in contrast to the ratio range in the reference group.

We divided the patients with NB into three groups due to uL-FABP/Cr ratio value: 1—with ratio in a range between 0 and 10 ng/mg; 2—between 11 and 100 ng/mg; 3—ratio greater than 100 ng/mg to assess factors which influenced the increase in the uL-FABP/Cr ratio. Significant differences between the above-mentioned groups in terms of the history of VUR, proteinuria in 24 h urine collection and urinary creatinine level were detected. More detailed data are presented in Table 3.

Overall, 27/42 (64%) of children with NB had no history either active or in the past, and their median uL-FABP/Cr ratio equaled 3.01 ng/mL (0.46–181.6 ng/mg). Only 3/42 (7%) of patients with NB had active VUR during our investigation with a median uL-FABP/Cr ratio of 14.37 ng/mg (13.63–15.83 ng/mg) and in the group of children with VUR diagnosed in the past that resolved the median uL-FABP/Cr ratio was 34.87 ng/mg (13.4 –214 ng/mg). Interestingly, all VURs either active or present in the patient’s medical history were unilateral and left-sided, but they differed in the grading. In children with NB and active VUR, 2/3 (66%) of VUR were low-grade (I°) and one was high-grade (IV°), whereas in children with NB and VUR in the past, all VURs were high-grade (IV° and V°).

In our study, a statistically significant positive correlation between uL-FABP/Cr ratio and proteinuria was revealed (r = 0.377, *p* < 0.05).

The majority of patients with NB—30/42 (%), had a lumbosacral spinal lesion, 7/42 (%) had thoracolumbar, and 5/42 (%) sacral level lesion. Of all the study subjects, 22/42 (52%) were classified as 1HS, 11/42 (26%) as 2HS, 6/42 (14%) as 3HS, and 10/42 (24%) as 4HS. We did not find statistically significant differences in the uL-FABP level between the above-mentioned groups, but we noticed statistically significant differences in the urinary creatinine (Chi^2^ = 11.59, *p* < 0.001) and the number of rUTIs (Chi^2^ = 19.2, *p* < 0.001) in Hoffer’s scale groups. Children from the 1HS group, described as wheelchair dependent, had the lowest urinary creatinine levels with a median of 42 mg/dL (16–102 mg/dL), and 77% (17/22) of them had a history of rUTIs, in contrast to children with different stages of walking impairment from remaining HS groups. In 2HS, the median urinary creatinine level was 109 mg/dL (45–200 mg/dL), and in 3HS it was 65.7 mg/dL (55.8–66 mg/dL), whereas in 4HS (described as community walkers), it was 64.5 mg/dL (25–137 mg/dL).

## 4. Discussion

This paper is a modest contribution to the ongoing discussions about renal biomarkers in NB population. The main purpose of the paper was to draw attention to the uL-FABP. This marker is located in the proximal tubular cells that may be the first localization of upper urinary tract injury in NB. The role of L-FABP excreted in the urine as a biomarker of tubular damage in children with NB has not been described.

Renal deterioration in the NB is usually an asymptomatic process. Several factors, including body muscle mass, can impact the rise in serum creatinine and decline in eGFR. Muscle wasting and impaired linear growth are observed in children with MMC due to denervation [13]. There may be substantial inaccuracy when we compare eGFR between patients with NB and the general population [14]. In our study, no correlations between anthropometric parameters and the uL-FABP were observed. It seems that the uL-FABP might be more reliable and does not depend on weight and growth by contrast with traditional kidney function markers, which is a precious quality of a renal deterioration biomarker in the NB population.

Our observations showed that the uL-FABP/Cr ratio was higher in patients with NB but the differences between the studied groups did not reach statistical significance. The most likely explanation of the negative result might be a small sample size. However, the range of ratio values was wider in NB in contrast to healthy participants. There are plenty of factors that determine the uL-FABP concentration. In our analysis, children with NB who had significantly elevated uL-FABP/Cr ratio (>100 ng/mg) tended to have lower urinary creatinine concentration, proteinuria in the 24 h urine collection and more frequent VUR diagnosis. Decreased urinary creatinine excretion and presence of proteinuria are well-known factors of progressive renal deterioration and predict greater risk of renal failure [15]. The increase in uL-FABP concentration may reflect the fact that the damage to the tubular cells has been done.

In our study, we assessed the effect of physical functioning, measured according to Hoffer’s scale, and uFABP concentration. The HS1 group was homogenous in terms of decreased physical activity, level of spinal lesion, and the worst functional health. They had the highest prevalence of rUTs that may give severe consequences, such as renal scarring and renal impairment. We hypothesized that uFABP will be elevated in wheelchair-dependent children (HS1) compared with different stages of walking impairment from the remaining HS groups. Surprisingly, there were no differences in uFABP concentration, as opposed to in urinary creatinine levels. We assume that the reduction in creatinine concentration in HS1 is due to a lack of physical activity and muscle wasting.

Our study revealed that proteinuria was positively correlated with the uL-FABP/Cr ratio. Under normal circumstances, proteinuria should not be detected in the final urine.

Pathological proteinuria may develop after dysfunction of the glomerular filtration barrier, impaired reabsorption of protein in the proximal tubule or both [16]. The uL-FABP is classified as a low-molecular-weight protein (14 kDa) that is detectable in the urine after the proximal tubule damage. The measurement of the uL-FABP may play a useful role in distinguishing glomerular from tubulointerstitial damage. However, higher concentrations of uL-FABP in NB patients from our research cannot be explained by proteinuria. Only 15/42 (36%) of children with NB had proteinuria. No statistically significant differences in urinary biomarkers in patients with and without proteinuria were observed (*p* = 0.56, *p* > 0.05).

The history of VUR is another factor affecting the uL-FABP concentrations. Very few publications are available in the literature that address the issue of VUR and the uL-FABP [17,18]. According to Benzer et al. [17], patients with active VUR showed a significant increase in uL-FABP levels compared to a reference group. Additionally, the uL-FABP levels were positively correlated with urinary protein excretion in children diagnosed with primary VUR. In our study, only 3/42 (7%) children from the NB group had active VUR, and they all had an elevated uL-FABP/Cr ratio in a range between 13.62 and 15.4 ng/mg. Parmaksız et al. [18] demonstrated that the uL-FABP/Cr level was higher in patients with renal parenchymal scarring due to VUR. However, studies on an association between the uL-FABP and VUR that had been resolved are still lacking. In our investigation, we have also considered the consequences of VUR in the past. The significant differences between the uL-FABP concentrations in children with and without a history of VUR were found. In our study, all VUR in the medical history of the enrolled patients with NB was high-graded and required surgical procedures. We assumed that increased the uL-FABP/Cr in these cases was associated with tubulointerstitial damage and fibrosis secondary to high-grade VUR in the past.

Children with NB need clean intermittent catheterization (CIC), increasing the risk of rUTIs [19]. This is a frequent complication that remains a challenge for specialists taking care of these patients. In our study, we did not observe higher L-FABP in children with NB with a history of rUTIs. This is a special feature and in contrast to other biomarkers, i.e., NGAL [20,21].

Our study has several important limitations. First, it is a single measurement of uFABP concentrations. The development of tubular damage and subsequent renal insufficiency will occur gradually over a long period. Measuring uFABP longitudinally in the same group of patients is crucial for determining its importance as an early biomarker of renal impairment. Based on the promising findings presented in this paper, work on the remaining issues is continuing and will be presented in future studies. Furthermore, this is a report based on findings from a single center. We still need multicenter research on uFABP in the neurogenic bladder to fully understand its role in tubular damage.

## 5. Conclusions

To conclude, we would like to highlight the following:

(1) L-FABP may be considered a potential biomarker of tubular injury in children with NB due to MMC.
(2) Proteinuria and history of VUR may be the factors influencing the uL-FABP concentrations.

However, further studies in the NB population are still warranted to gain further insights on uL-FABP.

## Figures and Tables

**Table 1:** Demographic characteristics of patients with NB and reference group.

Variables	NB Patients *n* = 42		Reference Group *n* = 18	*p* Value
Gender: Female/Male *n* (%)	24 (57)/18 (43)		15 (83)/3 (17)	0.06
Median (minimum–maximum)				
Age (years)	11 (2.75–18)		12 (0.67–18)	0.62
z-score height-to-age	−1.85 (−7–1.5)		0.3 (−3.3–2.6)	<0.001 *
z-score weight-to-age	−0.55 (−5–1.5)		−0.05 (−1.5–2.7)	0.06
z-score BMI (kg/m^2^)	0.45 (−4.2–2)		−0.1 (−3–2.9)	0.51
Serum creatinine (mg/dL)	0.37 (0.18–3.08)		0.52 (0.2–0.85)	0.03 *
Urinary creatinine (mg/dL)	50.1 (16–200)		98.3 (23.2–244)	<0.001 *
eGFR Schwartz (ml/min/1.73 m²)	77.3 (17–167)		117 (110–200)	<0.001 *

NB—neurogenic bladder; * *p* < 0.05.

**Table 2:** Biochemical parameters in patients with NB and reference groups.

Variables	NB Patients *n* = 42	Control Group *n* = 18	*p* Value
Urinary L-FABP (ng/mL)			0.14
(a) Mean	7.56	5.38	
(b) Median	1.96	4.15	
(c) Min—max	0.25–49.5	0.53–12.7	
Urinary L-FABP/Cr ratio (ng/mg)			0.52
(a) Mean	25.37	4.45	
(b) Median	4.77	3.75	
(c) Min—max	0.46–215	0.8–13.8	

Cr—creatinine.

**Table 3:** Comparison between L-FABP/Cr ratio groups in NB patients.

	I	II	III	Chi^2^	*p* Value
		L-FABP/Cr Ratio (ng/mg)			
	0–10	11–100	>100		
NB patients *n* (%)	27 (64)	11 (26)	4 (10)		
Proteinuria in 24 h urine collection (mg/24 h)	55	4.9	149.6	6.72	0.047 *
	(0–192)	(0–101)	(105–194)		
VUR *n* (%)				8.43	0.01 *
(a) without VUR	27 (100)	6 (54)	2 (50)		
(b) with VUR	0	3 (28)	0		
(c) VUR in the past	0	2 (18)	2 (50)		

NB—neurogenic bladder; VUR—vesicoureteral reflux; Cr—creatinine; * *p* < 0.05.

## Data Availability

The data presented in this study are available on request from the corresponding author. The data are not publicly available for ethical and privacy reasons.
